# COVID-19 and the Intersection of Race, Ethnicity, and Sexual Minority Status

**DOI:** 10.1093/geroni/igab046.891

**Published:** 2021-12-17

**Authors:** Robert Beringer, Brian de Vries, Gloria Gutman, Paneet Gill, Helena Dault

**Affiliations:** 1 University of Victoria, Victoria, British Columbia, Canada; 2 San Francisco State University, Palm Springs, California, United States; 3 Simon Fraser University, Vancouver, British Columbia, Canada; 4 Simon Fraser University, Surrey, British Columbia, Canada; 5 Victoria Hospice, Victoria, British Columbia, Canada

## Abstract

The COVID-19 virus has caused millions of deaths and impaired physical and mental health and social disconnection for countless persons around the world; concomitantly, the pandemic has exposed and exacerbated the pervasive effects of racism and stigma experienced by Black, Indigenous, or People of Color (BIPOC) and other marginalized/stigmatized groups. This study adopts an intersectional perspective examining multiple marginalized identities (i.e., the combination of LGBTQ and BIPOC status) and COVID-19 pandemic health stressors. We report on data from an online survey (conducted between Aug 10 and Oct. 10, 2020) focusing on current experiences and future planning during the COVID-19 pandemic in Canada. LGBTQ respondents (n=415) indicated significantly higher levels of depression, loneliness, sadness, and isolation in comparison to heterosexuals (n=3916). Heterosexual white respondents (n=3446) reported significantly higher levels of acceptance in their community and reported greater happiness but also higher rates of feeling of isolation than heterosexual BIPOC heterosexuals (n=470) who reported significantly higher rates of feeling judged/shamed by others than the heterosexual white respondents. In contrast to our expectations, white LGBTQ respondents (n=366) reported significantly more depression, loneliness, anxiety, and sadness than their BIPOC LGBTQ peers (n=49). These findings are interpreted as reflecting a complex mix of the effects of marginalization (as experienced by LGBTQ persons in general), and privilege and relative deprivation (as experienced by heterosexual and LGBTQ white persons) along with resilience and the moderated expectations and experiences of BIPOC LGBTQ persons.

